# Useless or used less? Poroscopy: The evidence of sweat pores

**DOI:** 10.1016/j.heliyon.2023.e17927

**Published:** 2023-07-03

**Authors:** Jaisleen Kaur, Meenal Dhall

**Affiliations:** Department of Anthropology, University of Delhi, Delhi 110007, India

**Keywords:** Fingerprints, Personal identification, Poroscopy, Sweat pores, Third level details

## Abstract

Poroscopy is the study of sweat pores present on the papillary ridges of the skin. This review paper aims to examine existing literature on poroscopy so that its relevance as a tool in personal identification can be established. Moreover, this paper aims to expound the various aspects of sweat pores as well as, highlight the contribution of poroscopy in latent, partial, and automated fingerprint matching. The relationship between sexual dimorphism, age, and sweat pores, effect of development technique and nature of surface on pore details, and use of sweat pores for liveness detection in biometric systems has also been explored. A review of all potentially relevant articles was conducted wherein, the non-relevant papers were excluded by screening their titles and abstracts following which, full-text review of all articles that met the inclusion criteria was carried out. The authors concluded that sweat pores present additional distinctive information for facilitating personal identification when used along with level 2 details. Furthermore, out of the various pore parameters namely, number, shape, size, inter-distance, position, and type, pore inter-distance was found to be most reliable.

## 1 Introduction

Fingerprints are frequently found evidences at the scene of crime (SOC) [1,2]. They are unique, immutable, and classifiable and act as an indispensable tool for personal identification [3]. Fingerprint matching can be done at three levels [[4], [5], [6], [7], [8], [9], [10], [11]]. The first level deals with macrofeatures [12] like pattern type, ridge count, core, delta, and orientation. Individualization cannot be achieved at this step. Level 2 comprises of comparing the relative nature and position of ridge characteristics, also known as Galton's minutiae, individualization can be achieved at this stage. Level 3 is based on the use of intra-ridge details [13] or microfeatures [14] like sweat pores, edge contours, friction ridge width, dots, incipient ridges, creases, and scars [8,12].

With the dismissal of numerical standards for achieving personal identification through the use of fingerprints, in countries like England, Wales, Scotland etc., greater flexibility has been granted to fingerprint experts in terms of which features to use while making identifications [15]. In other words, experience of the fingerprint expert along with the quality of features being examined, is given more importance than quantity thereby, allowing the use of extended features. Level 3 details encompass a significant subset of extended features, where pores are most prevalent [16].

Dr. Edmond Locard of Lyons, France developed the science of poroscopy in 1912 [6]. Sweat pores are the openings of sweat glands and function as a liquid-based cooling system that facilitates thermoregulation within the human body [17]. The process of sweat pore formation commences around the 14th week of gestation and culminates in complete development by the 24th week [18,19]. On average, there are approximately 2700 sweat pores per square inch of papillary skin [20]. According to Locard, pores, akin to ridge characteristics, exhibit permanence, immutability, and uniqueness. Furthermore, he proposed that a mathematical agreement between 20 and 40 pores is sufficient to establish the identity [6].

Poroscopy is especially useful when the recovered crime scene prints are blurred, partial, overlapping, have unspecified orientation, or contain low number of minutiae [8,16,[21], [22], [23], [24], [25], [26], [27]]. Locard obtained conviction, using poroscopy, in two landmark cases. In the Boudet and Simonin case, the crime scene prints lacked an overall pattern yet, 901 and 2000 sweat pores were found in the same relative location in the case of Boudet and Simonin, respectively. Similarly, in the Matern theft case, partial print recovered from the scene of crime containing 200 sweat pores was individualized as belonging to the right ring finger of a burglar named Matern [6].

Sweat pores can be analysed on the basis of number (per unit area and per unit length), shape, size, position (middle or periphery), type (closed, open on one end, and open on both ends), and inter-distance. The following sections will focus on these aspects of sweat pores.

## 2 Materials and methods

Google Scholar and PubMed databases were used to search for articles on poroscopy using the keywords: ‘poroscopy’, ‘poroscopy forensic science’, and ‘poroscopy biometrics’. Articles in English language pertaining to the use of sweat pores in the field of forensic science, biometrics and/or personal identification were included. Articles were primarily screened using their title and abstract. Relevant articles were full-text reviewed. To ensure that no relevant research paper was left out, articles from back references of included research papers were also screened. All papers thus yielded were included. A total of 60 research papers were obtained, after eliminating duplicate search results, and reviewed. Table 1. Summarizes the major research findings of some studies included in this review paper.

**Table 1:** Major research findings of some included studies.

Authors(s)	Findings
Faulds (1913)	- Pores remain locally fixed in nature - Pore shape and size is not reproducible
Ohler & Cummins (1942)	- On an average, females possess 23.4 ± .07 ridges per cm while, males possess 20.7 ± .06 ridges per cm - Females possess significantly finer ridges than males
Weisenberg et al. (1975)	- Significantly higher number of sweat pores in females than in males - Significantly higher number of open sweat pores in females
O'Leary et al. (1986)	- 20.7 pores per cm of dermal ridge
Andersen & Pedersen (1987)	- Presence of significantly less number of sweat pores per unit area in males as compared to females - No difference in the number of sweat pores on the right and left hand - No correlation between age and number of sweat pores for any finger - Higher number of sweat pores per unit area on digits III and IV as compared to digit I, II, and thumb
Scobbie & Sofaer (1987)	- Number of sweat pores per cm of friction ridge showed a decrease with increasing age (cross-sectional study) - Negative correlation was found between hair density and number of sweat pores thereby, implying that the same genes influence the two traits in opposite directions - A minute but statistically significant laterality effect was observed between the digits of the left and right hand
Stosz & Alyea (1994)	- False accept rate of 0.04% and false reject rate of 6.96% was achieved by combining minutiae and pore information
Osten et al. (1998)	- Positively recognized fake finger attacks in biometric systems using ECG, temperature, and pulse of user
Ashbaugh (1999)	- Pores exist in a variety of shapes - Position of pores on the ridge is random - Small pores are more common as compared to large pores - Pore location is immutable
Acree (1999)	- Females possess a significantly higher ridge density as compared to males - Fingerprint containing ≤11 ridges/25 mm^2^ is most likely to belong to a male - Fingerprint containing ≥12 ridges/25 mm^2^ is most likely to belong to a female
Bindra et al. (2000)	- 8 to 25 pores per cm of friction ridge - More pores were observed in the lateral hypothenar and digital pad area as compared to the thenar region - Medium-sized pores were most commonly found followed by minute- and large-sized pores - Rhomboid-shaped pores were most common (29%–39%) followed by round (23%–31%), elliptical (12%–25%) and rectangular-shaped pores (15%–19%) - Closed pores were more common (65%–80%) than pores open on one and both sides - Ninhydrin developed prints on paper with exceptional pore quality - Iodine fuming was found to be suitable for both porous and non-porous surfaces - Silver nitrate produced good quality pores on paper
Kiss (2001)	- Successfully developed a fingerprint biometric system that recognized life signs of users based on electrical conductivity of the skin
Matsumoto et al. (2002)	- Moisture was found to be 16% and 23% for live and gelatin fingers, respectively - *Gummy* fingers were accepted with probability of 68–100% in 11 different fingerprint recognition systems
Kryszczuk et al. (2004)	- Depicted that use of fingerprint fragment containing level 3 features, instead of the entire fingerprint, produced equally reliable recognition results
Parthasaradhi (2005)	- Distinctive spatial moisture pattern was observed in live fingers - Classification rate was found to be 80–93.33% and 80–95% by capacitive dc device for live and spoof fingers, respectively - Classification rate was found to be 62.5–93.3% and 81–100% by electro-optical device for live and spoof fingers, respectively - Classification rate was found to be 73.3–100% and 85.7–95.4% by optical device for live and spoof fingers, respectively
Moon et al. (2005)	- Surface coarseness was used to detect fingerprint liveness
Gupta et al. (2007)	- %C.V. (Coefficient of Variance) was found to be outside acceptable limits thereby, putting the reliability of pore size and shape in doubt - No surface (i.e., 80 gsm white paper, copier laser jet paper, 90 gsm laser paper, 160 gsm laser jet paper, 160 gsm pulp board paper – Hewlett Packard USA, 260 gsm matt inkjet paper, 106lb 100% cotton paper, acid free paper – Strathmore paper mill Franklin, 260 gsm gloss paper, ink jet paper – Jessops photo England, invoice paper, paper used for National Fingerprint Form, glass plates, glass slides, and transparency sheets) was found to be reliable for the measurement of surface area in inked prints - Non-absorbent surfaces (glossy papers, transparencies, and glass slides) were found to reveal more pore detail as compared to non-glossy papers
Choi et al. (2007)	- Individual pore spacing, noise, and first order statistics were successfully used to calculate the liveness score of fingerprint images captured using NITGEN fingerprint sensor at 500dpi resolution, in order to detect fake fingerprints - Individual pore spacing was analysed using correlation filter method
Jain et al. (2007)	- Pores and ridge contours were extracted from 1000 dpi images using wavelet transform and Gabor filters followed by matching using modified ICP algorithm - Significant performance improvement (20%) was observed when the proposed Level 3 matcher was combined with Level 2 matcher
Gutiérrez et al. (2008)	- Fingerprint ridge density was found to be significantly higher in the radial and ulnar areas of all fingers in females as compared to males - Ridge density was less in the radial and ulnar areas of the index finger and thumb, in both the sexes, as compared to the middle, ring, and little finger - Ridge density was found to be higher in the lower area of the thumb and index finger as compared to the middle, ring, and little finger in both the sexes
Chaberski (2008)	- Successfully developed a feature extraction software that assessed, matched, and evaluated five pore attributes namely, quantity, size range, average pore size, average pore/ridge contrast, and average outline strength and four ridge contour characters i.e., quantity, average ridge/valley contrast, distance between detected core and image center, and ratio of ridges to valleys
Reddy et al. (2008)	- Oxygen saturation used for detecting life signs of the user
Gupta & Sutton (2009)	- Pore area was found to be reproducible within a batch of direct images taken over a single hour on the same day - Pore area was not found to be reproducible in direct microscopic images captured on different days - Deposition and development technique made a significant contribution to the pore area variability
Singh (2009)	- Fingerprints developed with printer's ink were much superior to those developed using pre-inked fingerprinting pads in terms of clarity of pores
Zhao et al. (2010)	- Increase in false detection rate was observed with a decrease in fingerprint image quality - Decrease in fingerprint image quality had a direct effect on the pore matching accuracy of both the proposed pore matcher and VeriFinger - Fusion of pores with VeriFinger minutiae matcher was found to improve the recognition accuracy in low quality fingerprint images - Recognition accuracy of the minutiae-based matcher improved significantly when the fingerprint image resolution increased from 500 ppi to 1000 ppi however, improvement in the accuracy of pore-based matcher was relatively small
Malathi & Meena (2010)	- Pore-based Local Binary Pattern used to successfully match partial fingerprints against full fingerprints
Zhao et al. (2010)	- High resolution partial fingerprints matched and aligned against full fingerprints using a novel pore-valley descriptor that detects, extracts, and utilizes pore location and orientation
Oklevski (2011)	- Spatial arrangement of pores was found to be stable - Number of pores per unit distance remains stable
Anthonioz et al. (2011)	- Pore location was found to be reproducible whereas, pore size and shape showed weak reproducibility in images recorded using coaxial illumination system coupled with an SLR camera - Latent fingerprints developed using cyanoacrylate fuming showed better third level characteristics as compared to those developed using ninhydrin and DFO - Non-porous surfaces exhibit better third level characteristics
Nagesh et al. (2011)	- 5–16.3 pores per cm of ridge in males - 5.3–16 pores per cm of ridge in females - Closed pores were most commonly found followed by pores open on one side and pores open on both sides - 69–284 μm pore size in males - 66–287 μm pore size in females
Preethi et al. (2012)	- 7.83 pores per cm of ridge in males - 9.36 pores per cm of ridge in females - Fingerprint containing ≤8 pores per 25 mm^2^ more likely to belong to a male - Fingerprint containing ≥9 pores per 25 mm^2^ more likely to be of female origin
Agnihotri et al. (2012)	- 8–14 ridges/25 mm^2^ in males - 10–20 ridges/25 mm^2^ in females
Eshak et al. (2013)	- Females possess statistically significant (i) narrower finger breadth, (ii) smaller square area, (iii) higher ridge count, and (iv) higher ridge density, as compared to males
Lee et al. (2014)	- New hydrochromic sensor system developed for mapping human sweat pores
Oktem et al. (2015)	- Fingerprint containing ≥15 ridges/25 mm^2^ in the radial or ulnar region and ≥13 ridges/25 mm^2^ in the inferior region is more likely to belong to a female
Park et al. (2016)	- Four Hydrochromic systems namely: Hygroscopic headgroup Polydiacetylene (PDA), Inkjet compatible and temperature dependent hydrochromic PDA, Fluorescein hydrophilic polymer poly(vinylpyrrolidone) (PVP), and Carbon nanodots were successfully used to map sweat pores on human fingertips
Monsoon et al. (2019)	- Level I details were found to be both permanent and persistent in short- as well as, long-term study - Creases were not found to be permanent in long-term study - Level II details were found to be both permanent and persistent in short term study period however, they were persistent but not permanent in long-term study period - Size, shape, and presence of sweat pores was neither permanent nor persistent in both short- as well as, long-term studies - Pore location was found to be same
Sharma et al. (2019)	- Significant correlation was observed between the number and position (middle) of pores on the ridge of the thumb and ring fingers - Significant correlation was observed between the number of pores and position (periphery) of pores on the ridge of the index and ring fingers
Kaur & Dhall (2022a)	- Study highlighted the reproducibility and reliability of sweat pore inter-distance and angle in inked fingerprints - Pore inter-distance and angle were observed to be reproducible and thus, can be utilized for fingerprint matching - Better quality of pores was visible in the case of fingerprints recorded on the sticky-side of adhesive tape
Kaur & Dhall (2022b)	- Pore inter-distance and angle were reproducible, even when control fingerprints were matched with corresponding specimen fingerprints obtained after immersing the hand in water at different temperatures for various time intervals - Study encouraged the use of pore inter-distance and angle for matching fingerprints, in conjunction with level 2 details

## 3 Various aspects of sweat pores

### 3.1 Number of pores

The number of pores can be calculated in two ways i.e., average number of pores per unit length and per unit area. The number of pores was observed to be 9–18 and 20.7 per cm of epidermal ridge by Locard [6] and O'Leary et al. [28], respectively. Similar findings were observed by Bindra et al. [29] wherein, 8–25 sweat pores were observed per cm of friction ridge. Interestingly, the number of sweat pores per unit distance has been reported to be stable with time [21].

Additionally, a minute but statistically significant laterality effect has been observed between the digits of the left and right hand i.e., digits of the left hand, on an average, possess 0.29 sweat pores/cm more than that of the right hand, in both the sexes [30]. This finding, however, was contradicted by Andersen and Pedersen [31].

### 3.2 Shape

Pores exist in a variety of shapes like round, oval, elliptical, triangular, rhomboid, and square [6,29]. In a study conducted by Bindra et al. [29], rhomboid-shaped pores were most abundant followed by round, elliptical, and rectangular pores. However, more recent studies [26,32] suggest that round and oval [25] pores are more common whereas, rhomboid and rectangular are less common pore shapes. Please note that a sweat pore is an oval- or round-shaped entity however, adherence of ink on the pore margin and subsequent surface deposition is responsible for giving it a triangular, rhomboid, rectangular, oval, or elliptical appearance. In simple words, sweat pores are circular or oval however, they may appear to be triangular, rectangular, rhomboid etc. owing to the adherence of ink/fingerprint powder on their margins.

Furthermore, pore shape is not a reproducible feature [10,15,16,33] as, distortions caused due to pressure change the basic shape of pores [6].

### 3.3 Size

Various studies [6,14,15,25,26,29,32,34] have focused on the size of sweat pores. Pores ranging from 88 to 220 μm in diameter were first reported by Locard [6], similar findings were observed by Roddy and Stosz [35] wherein, the size of pores was found to be 88–200 μm in diameter. More recent studies have reported pore size (diameter) to be 265 μm [25], 69–284 μm in males, and 66–287 μm in females [26].

Different-sized pores can be observed on the same ridge in no particular sequence [6,29]. Medium-sized pores are most common, followed by minute-sized pores whereas, large pores are most unusual [6,26,29]. Studies [10,[14], [15], [16],^25^,^33^,^36^] have revealed that the size of pores is not reproducible. Moreover, changes in the secretory activity of sweat pores, studied using hydrochromic sensors [23,24] and fluorescein-polyvinylpyrrolidone (PVP) composite film [37] suggest that the size of pores keeps on changing based on their physiological activity [34] and cannot be considered reliable while matching two fingerprints.

### 3.4 Position of pores on the ridge

**Fig. 1:**
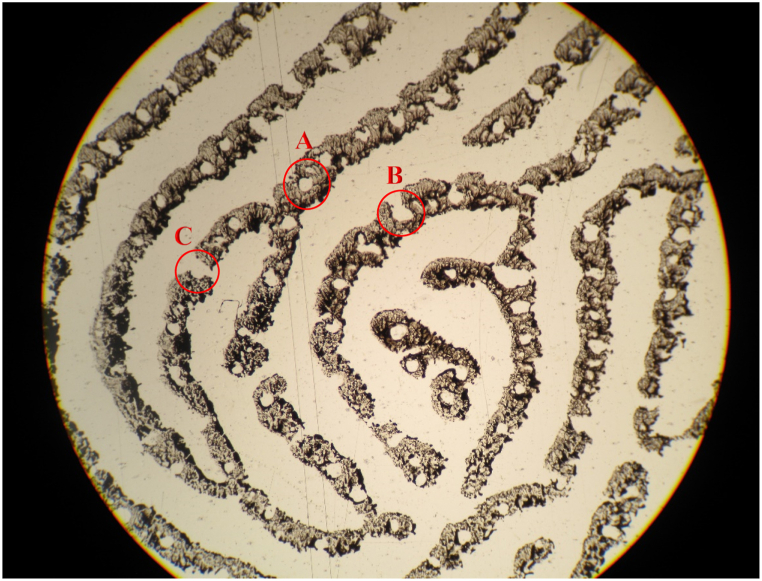
Photomicrograph captured at × 40 magnification using Zeiss Primo Star microscope attached with Canon PowerShot G10 camera showing different types of pores: (A) closed pore, (B) pore open on one end, and (C) pore open on both ends.

Sweat pores may be positioned near the periphery or middle of the papillary ridge [6]. When located in the middle (of the ridge), they are closed whereas, when present towards the periphery, they may be open on one side or both sides (see Fig. 1). Closed pores are more frequently found as compared to pores open on one side and both sides [26,29,32]. It is crucial to understand that it is not the sweat pore which is open or closed instead, the adherence of ink on the pore margin gives it such an appearance. In simple words, a sweat pore is a closed entity which may appear to be ‘open on one side or both sides’, owing to the adherence of ink. With change in pressure and angle during print deposition, consistency of ink, and method of print development, the type of pore (closed, open on one side, and open on both sides) will also change. Thus, one must not expect to find the same pore obtained from the same finger of the same individual, to be exactly alike in this respect.

A significant correlation has been observed between (i) number of pores and middle position for the thumb and ring finger and (ii) number of pores and periphery position for the index and ring finger [38].

### 3.5 Interspacing

**Fig. 2:**
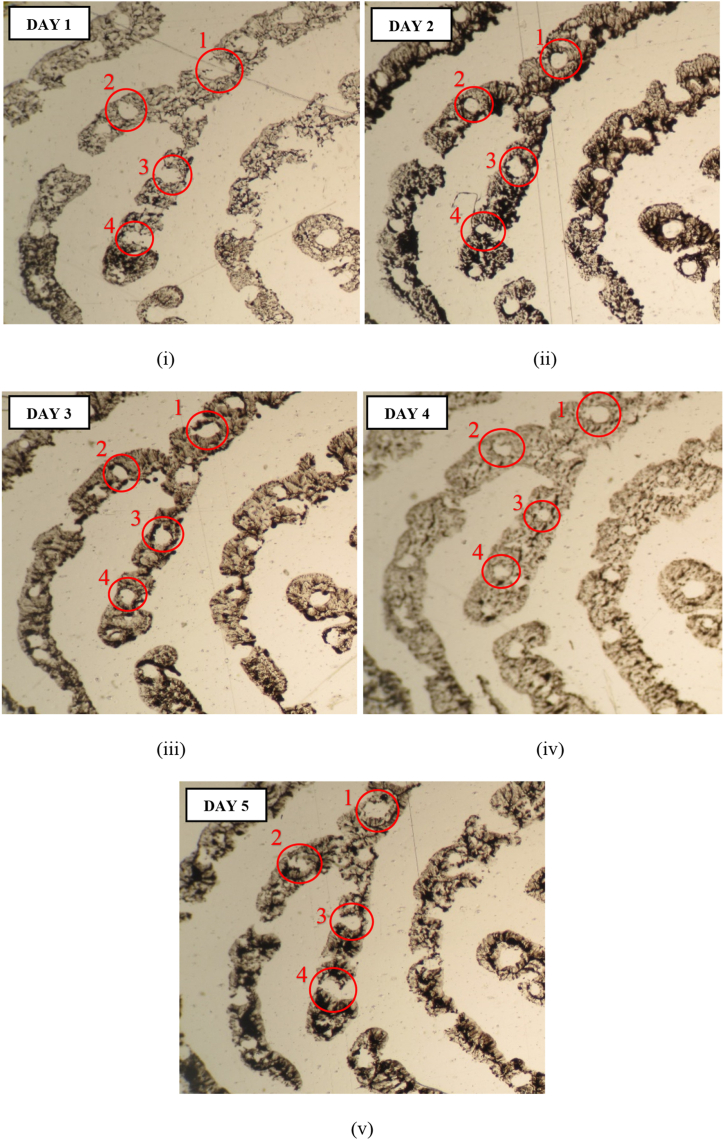
Photomicrographs (i–v) of inked fingerprints obtained from the same finger of the same individual, captured at × 40 magnification using Zeiss Primo Star microscope attached with Canon PowerShot G10 camera, showing variations in the size, shape, and position (closed, open on one side and both sides) of four pores over five consecutive days.

When a pore undergoes distortion, it stretches (owing to elasticity and compression of the skin), which results in change in its shape, size, and type (see Fig. 2). However, the inter-distance remains more or less the same i.e., within acceptable reproducible limit (with 5% confidence level in analytical work) [36]. Furthermore, it has been observed that in case of pressure distortions involving major ridge path deviations, pores remain in the same relative position to each other during flexion [6]. Thus, pore inter-distance, from the centre of one pore to the centre of the adjacent pore, is reproducible for every individual. Therefore, connecting the centres of sweat pores, on the same ridge, will generate a pattern which is distinct for every individual [6]. Alternately, connecting the centre of a pore to the centre of another pore on a parallel ridge will also result in a unique pattern [39]. Studies [[22], [23], [24],^36^,^37^,^40^,^41^] have demonstrated the reliability of pore interspacing, while, studies [6,29,34,39] suggest the use of pore inter-distance for individualizing fingerprints. Furthermore, the probability of occurrence of 20 intra-ridge consecutive pores in the same configuration is 1.16 × 10^−14^ [35] thereby, implying the individualistic nature of sweat pores, in terms of pore location.

Oklevski [21] studied pores in 100 pairs of inked fingerprints, taken over a time period of 5–48 years, and found the spatial arrangement of sweat pores to be stable in samples even when the time interval in dactyloscopying was 48 years. Additionally, it was observed that two non-identical fingerprints with four matching ridge characteristics, differed completely in terms of spatial arrangement of pores. Similar findings were observed by Monson et al. [33] wherein, sweat pores were found to be persistent in location even after an elapsed time of 29 years.

In work conducted by Anthonioz and Champod [42], high resolution (∼2700 dpi) images recorded using coaxial illumination system were used to study pore configurations relative to a reference point (ridge characteristic), in an attempt to distinguish between same-source and different-source fingerprints. Although images of such high quality are not retrieved from the SOC, the study was carried out to assess the contribution of sweat pores at high(est) level of clarity so that, the results can aid fingerprint practitioners in assessing the strength of evidence associated with pores. It was observed that pores when considered in combination with ridge characteristics provide good discrimination ability.

Grey level extraction of pores using principal component analysis (PCA) of *putative* pores was carried out by Parsons et al. [40] wherein, the authors were able to conclusively determine whether the pore patterns originated from the same or different finger (a match between two pores was declared only if they were less than about 10 pixels apart). Cai et al. [22], successfully used level 3 features in combination with the chemical information obtained from fingerprints to positively establish the identity.

Sweat pore mapping using blue-to-red transforming polydiacetylene(s), containing Caesium carboxylate head group(s) (PDA-Cs) film, was carried out by Lee et al. [23] and Park et al. [24] wherein, the polymer film turned red instantly upon coming in contact with the fingertip. Scrutinizing the area of contact revealed numerous red-coloured dots. A superimposition-based pore-pattern-matching program, using Image J and MATLAB, was implemented in which the image containing red microdots was compared with a ninhydrin-developed latent fingerprint image of the same finger. The fingerprints were found to match (overlap) precisely thereby, demonstrating the reliability of pore interspacing. In a study by WooáLee and JunáPark [37], water-responsive fluorescin-PVP composite film was used for sweat pore mapping. This method, like [23,24], enabled colorimetric differentiation between sweat secreting active and inactive pores. Moreover, it demonstrated the reliability of pore interspacing.

## 4 Sexual dimorphism, age, and sweat pores

Determination of sex and age from fingerprints can help in narrowing down the list of possible suspects. Various attempts [26,30,31,32] have been made to establish whether or not there exists any relationship between the number, shape, and position of pores and sex and age of an individual.

It has been observed that females, as compared to males, possess a significantly higher number of sweat pores per unit area [31,32,43] and per unit length [30] of friction ridge skin (FRS). This can be attributed to the presence of finer and higher number of epidermal ridges in females as compared to males [[44], [45], [46], [47], [48], [49]]. The average number of sweat pores per cm of ridge have been observed to be 8.40 [26] and 7.83 [32] in males and 8.83 [26] and 9.36 [32] in females. Furthermore, in a study conducted by Dasa et al. [32], a fingerprint containing ≤8 pores/25 mm^2^ was more likely to belong to a male whereas, that containing ≥9 pores/25 mm^2^ was more likely to belong to a female.

On the contrary, number of pores was not found to be statistically significant in the two sexes, in studies by Nagesh et al. [26] and Scobbie and Sofaer [30]. Additionally, no correlation was observed between age and number of sweat pores for any finger by Andersen and Pedersen [31].

## 5 Effect of development technique and nature of surface on pore details

Clarity of pore details in latent fingerprints is directly dependent on the nature of substrate and method of development [15]. Sweat pores are better revealed in latent fingerprints developed using chemical methods like ninhydrin and silver nitrate as compared to fingerprint powders [4]. Non-porous surfaces like glass slides, transparency sheets, and glossy paper exhibit better pore details in contrast to porous surfaces like paper [10,15]. This can be due to the presence of fibres in the latter which introduces distortion thereby, changing the pore shape [15]. Additionally black printer's ink has been found to be superior in developing pore details as compared to pre-inked fingerprinting pads [39].

In case of non-porous surfaces (like mica sheets, glazed metal sheets, and glass), cyanoacrylate fuming [10] has been found to be most suitable while, iodine fuming [29] has shown to give satisfactory results for both porous and non-porous surfaces. Additionally, high clarity pores on paper have been observed by ninhydrin and silver nitrate [29]. Pore visibility was found to be minimum in latent fingerprints developed on paper using 1,8-Diazafluoren-9-one (DFO) [10].

## 6 Automated fingerprint matching using pores

Fingerprints are widely used as biometric identifiers [33,50]. However, it has been observed that majority of automated fingerprint identification systems employ only level 1 and 2 attributes of fingerprint matching whereas, level 3 details are rarely used [7,[11], [12], [13],^16^,^27^,^51^,^52^]. This is because traditional fingerprint 500 dpi images do not usually contain level 3 features of adequate integrity [12,53]. However, with the development of high-resolution fingerprint scanners extracting richer level 3 details from fingerprint images has become possible [12,16]. Minimum scanning resolution of 1000 ppi (for capturing latent, ten print, and palm print images) and inclusion of level 3 features in the Federal Bureau of Investigation (FBI) standard, was proposed by ANSI/NIST fingerprint standard update workshop (American National Standards Institute, National Institute of Standards and Technology) in 2005 [54]. Additionally, numerous studies [9,10,12,13,16,27,35,42,52,53] suggest incorporating third level details in fingerprint identification and matching systems in order to increase efficiency and reduce error rates.

### 6.1 Complete fingerprint images (captured using live scanners)

Livescans produce immediate digital images and can help in prompt collection of fingerprints [33]. First fingerprint matching algorithm using sweat pores was proposed by Stosz and Alyea [9]. It involved converting a grey scale image from the sensor into binary format followed by processing the binary image till a skeleton image was obtained. This skeleton image was processed further to improve its functionality from a ridge characteristic analysis viewpoint (image quality was enhanced by eliminating “ridge noise” followed by syntactic processing or “healing”). False accept rate (FAR) of 0.04% and false reject rate (FRR) of 6.96% (compared to ∼31% for minutiae alone) was achieved.

A study to mathematically demonstrate the uniqueness of pores, was conducted by Roddy and Stosz [35], wherein the following observations were made, (i) probability of two consecutive intra-ridge pores having the same relative spatial location with two other pores was 0.04, (ii) probability to have 20 intra-ridge consecutive pores in the same configuration was found to be 1.16 × 10^−14^, and (iii) probability of occurrence of a particular combination of 20 ridge independent pores was 5.186 × 10^−8^. The major limitations of these studies [9,35] were (i) limited database, (ii) computationally expensive, (iii) only high quality images were used for effective pore extraction, (iv) only custom-built optical sensors of ≥2000 ppi were used (instead of commercially available live scan sensors of 1000 ppi), and (v) manual alignment of the query and template fingerprint fragments.

In order to overcome the aforementioned limitations, a fully automatic fingerprint matching system, employing wavelet transform and Gabor filter enhancement along with Iterative Closest Point (ICP) algorithm, to solve the problem of non-linear deformation and image degradation, was employed by Jain et al. [12] wherein, 1640 fingerprint images (1000 ppi) acquired using a commercial optical live scanner (CrossMatch 1000ID) were analysed. A significant performance gain of ∼20% across different image quality groups, when combining level 2 and level 3 features in a hierarchical manner, was observed as against level 2 matcher alone.

### 6.2 Latent fingerprints

Zhao et al. [16] proposed a feature matching algorithm based on level 3 details, to match latent to full fingerprints, based on dots, incipient ridges, and ridge edge protrusion (DIP) utilizing the topological relationship between level 2 and 3 details, to eliminate the effect of non-linear distortion. An increase in the latent fingerprint matching accuracy from ∼26.9% to ∼31% was observed when both the latent and fingerprint exemplars were of good quality. In a similar study [55], the utility of sweat pores in the case of varying fingerprint image quality and resolution was studied using a commercial-off-the-shelf (COTS) minutiae matcher (VeriFinger) and a pore-based matcher, in rolled fingerprint images of resolution 500 dpi and 1000 dpi. Increase in false detection rate and decrease in pore recognition accuracy (for both VeriFinger and pore matcher) was observed with a decrease in fingerprint image quality thereby, signifying the direct relation between quality of fingerprint image and efficiency of AFRS in its ability to extract and match pores.

### 6.3 Partial fingerprints

Fragmentary latent prints bearing low number of minutiae but ample level 3 details should never be discarded [16]. An attempt to investigate the relationship between fingerprint fragment size and the distinction potential of minutiae and pores was made by Kryszczuk et al. [13] wherein, reducing the size of test fragment decreased misalignment thereby, facilitating the recognition process (provided that the test fingerprint is of high quality).

In a study conducted by Chaberski [53], high resolution fingerprint images of 2000 dpi, with physical area of 0.27 × 0.34 inches (partial prints), were matched against full fingerprint images using various pore and ridge attributes namely, quantity, size range, average pore size, average pore/ridge contrast, average outline strength, distance between the detected core and image centre, and ratio of ridges to valleys. It was observed that although, with reference to sweat pores, matching performance was highly dependent on the quality of fingerprint template, hierarchical method using both level 2 and 3 features improved the overall EER by reducing the false non-match rate (FNMR).

Attempts at matching partial fingerprints against full fingerprints using local binary pattern algorithm [56] and pore valley descriptors [27] were carried out. The major advantage offered by these algorithms was that they were not dependent on the minutiae matching results in order to match pores.

## 7 Liveness detection

With advancements in science and technology and enhanced level of awareness among criminals, fake fingerprint attacks have become quite common [57]. Present day multispectral sensor systems can be deceived by using fake fingerprints [58]. Acceptance rate of artificial gelatin fingers, in 11 different fingerprint recognition systems, was studied by Matsumoto et al. [57] wherein, *gummy* fingers were accepted (in all the 11 systems) with a probability of 68–100%. Thereby, focusing on the need to develop biometric systems which can efficiently detect life signs (aliveness) of users.

Several attempts have been made to recognize fake fingers these include, detecting electrocardiogram (ECG), temperature and pulse [59], impedance, electrical conductivity [60], texture coarseness [61], and oxygen saturation (SpO_2_) [62] of the user. However, most of these systems are not available commercially, have not been tested and evaluated rigorously (for user acceptance, universality, collectability, false accept and reject ratios), and require additional hardware to capture the liveness features, making them expensive and bulky [63].

Vitality detection [64], whether the introduced biometric is from a live source or not, using perspiration pattern of sweat pores was studied by Parthasaradhi et al. [63] wherein, a *distinctive spatial moisture pattern which evolves in time due to the physiological perspiration process* was observed in 33 live fingers, as against 14 cadaver, and 33 spoof fingers, using fingerprint scanners with no additional hardware requirement. The classification rate for live and spoof fingers was observed to be 80–93.33% and 80–95%, 62.5–93.3% and 81–100%, and 73.3–100% and 85.7–95.4% by capacitive dc, electro-optical, and optical scanners respectively, using an image-capture window of 2 seconds. Fake finger detection based on skin elasticity was proposed by Antonelli et al. [65] wherein, sequential fingerprint images captured when the user rotates his/her finger on the sensor, are used to calculate the distortion code. A novel multiple static feature-based aliveness detection method, pertaining to the physiological and statistical characteristics of live and fake fingerprints such as pore spacing, residual noise, and several first order statistics, was developed by Choi et al. [50] wherein, classification rate of 85% was observed by using only a single image. New optical methods for liveness detection based on analysis of (i) pulse using movement of papillary lines of the fingertips, (ii) change of colour and elasticity of the fingers due to pressing against a glass plate, and (iii) light illumination of the fingertip at different wavelengths (contactless liveness detection method) was proposed by Drahansky et al. [66].

Furthermore, certain studies [67,68] used optical coherence tomography (OCT), a non-invasive technique, to capture high-resolution three-dimensional fingerprints [67,68]. This method not only provided more information as compared to conventional two-dimensional fingerprints but also, was able to successfully distinguish between real and fake fingers (as fake fingers did not possess internal structures).

## 8 Limitations and suggestions

For better understanding of poroscopy, a longitudinal study focusing on the various aspects of sweat pores should be carried out, on different surfaces, using different physical and chemical methods of fingerprint development. Secondly, a consensus on how to mark level 3 features should be reached among fingerprint experts in order to increase their utility in fingerprint matching.

Most studies have focused on plain left thumb prints for scrutinizing the shape, size, number, position, and interspacing between pores. Future research work should focus on prints from the palmar region as well as, plain and rolled fingerprints of all ten digits obtained on various porous (paper, cardboard etc.), semi-porous (latex gloves etc.), and non-porous surfaces (metal, plastic, cellophane, tape, transparency sheets etc.), using different techniques such as fingerprint powder dusting, iodine fuming, ninhydrin, silver nitrate, small particle reagent (SPR), phase transfer catalyst (PTC), cyanoacrylate fuming etc., for studying the pore details. Additionally, pore features should also be studied in fingerprints deposited at different pressures (high, medium, and low), distortions, and weather conditions (temperature, humidity etc.). Such work will not only generate ample data but will also help give a better understanding of sweat pores. Moreover, if such studies are carried out using large sample sizes in different parts of the world, morphological variations, if any, in sweat pores among different populations can be studied.

A major limitation of poroscopy is that sweat pores may not always appear in inked, latent or livescan fingerprints (poor quality livescan images are often obtained in case of users with dry skin). Though, amount of pressure applied while depositing the print, nature of surface, presence of contaminants (like dust), type of development technique used, uniformity of ink etc. are amongst the plausible reasons behind this, another possible factor can be that not all crime scene personnel/fingerprint experts are aware about poroscopy and its scope as a tool in personal identification, as a consequence of which, proper precautions (with reference to pores) are not taken while developing latent prints at the SOC. This often leads to pore fill-ins and thus, a reliable method of identification slips into oblivion. Adequate knowledge of poroscopy along with careful dusting of latent prints (in order to avoid pore fill-ins), recording of good quality inked and full fingerprint exemplars, use of moisture-free fingerprint powder, and brushing in the direction of ridge flow are some ways by which the aforementioned can be rectified.

Studies focusing on the inter-print variations between direct microscopic images, reference prints, and latent prints developed by various methods on different surfaces should be conducted to supplement the existing literature.

## 9 Discussion

Although, the technology used to develop crime scene fingerprints has improved to the point where pores appear more frequently in ridges, poroscopy is accepted in theory but ignored in practice [6]. Fingerprint examiners seldom use third level details due to the absence of set guidelines moreover, no clear consensus on their classification, reproducibility, and individual value exists among practitioners [69].

This review paper highlights the importance of poroscopy as a method in personal identification. After reviewing all relevant research papers (as yielded by the search criteria), the authors are of the opinion that out of the various pore parameters namely, number, shape, size, inter-distance, position, and type, pore inter-distance (location) is most reliable.

Poroscopy can be efficiently used in automated fingerprint matching using livescan fingerprint images, of both high and low quality. Significant improvement in EER was observed while combining level 2 and level 3 characteristics, in a hierarchical manner, as compared to level 2 alone [9,12,52]. Moreover, the common assertion, that level 3 features can only be used when the fingerprint image quality is high, was not found to hold good as, consistent performance gain was observed across varying image quality groups. However, in the case of partial and latent fingerprints, pore extraction and resulting matching accuracy were found to be significantly dependent on the fingerprint image quality of the exemplars and latent/partial prints thereby, emphasizing on (i) careful dusting/development of latent prints (in order to avoid pore fill-ins) and (ii) recording good quality inked and livescan exemplars.

## 10 Conclusions

The science of poroscopy is largely unexplored. The same can be understood by the search results, which show a dearth of available research data on poroscopy thereby, emphasizing on the need to conduct more work in this field.

Forensic science is the science for justice and evidence, no matter how minute, should never be overlooked. Sweat pores are a part of the valid information provided by fingerprints and should not be ignored. They are difficult to mimic, present in abundance, and can act as a competent tool for facilitating personal identification, when used along with ridge characteristics.

## Author contribution statement

All authors listed have significantly contributed to the development and writing of this article.

## Data availability statement

Data included in the article is available with the authors.

## Additional information

No additional information is available for this paper.

## Declaration of competing interest

The authors confirm that this review article is an original work and has not been presented or published elsewhere nor is under consideration for publication elsewhere. The authors declare that they have no conflicts of interest. Both authors have approved the manuscript for submission.
